# Supplementary material for: PAP8/pTAC6 Is Part of a Nuclear Protein Complex and Displays RNA Recognition Motifs of Viral Origin
Source: Int J Mol Sci. 2022 Mar 11;23(6):3059. doi: 10.3390/ijms23063059 (PMC8954402; doi:10.3390/ijms23063059)
Supplement: Supplementary file 1 [file ijms-23-03059-s001.zip › ijms-1600905-Supplementary Mat/Figure S1.pptx]

## Slide 1
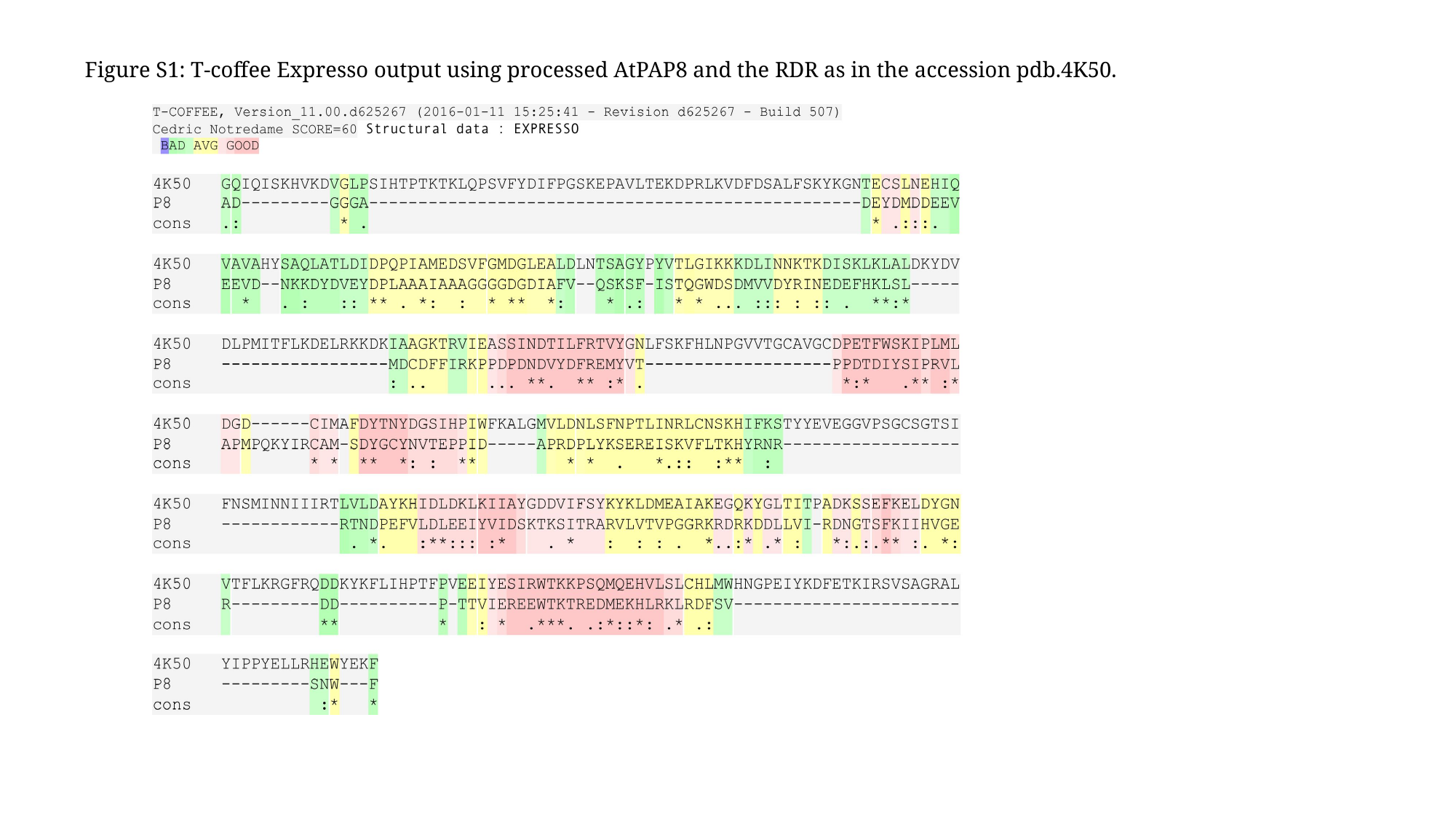

Figure S1: T-coffee Expresso output using processed AtPAP8 and the RDR as in the accession pdb.4K50.
