# Supplementary material for: PAP8/pTAC6 Is Part of a Nuclear Protein Complex and Displays RNA Recognition Motifs of Viral Origin
Source: Int J Mol Sci. 2022 Mar 11;23(6):3059. doi: 10.3390/ijms23063059 (PMC8954402; doi:10.3390/ijms23063059)
Supplement: Supplementary file 1 [file ijms-23-03059-s001.zip › ijms-1600905-Supplementary Mat/Figure S2.pptx]

## Slide 1
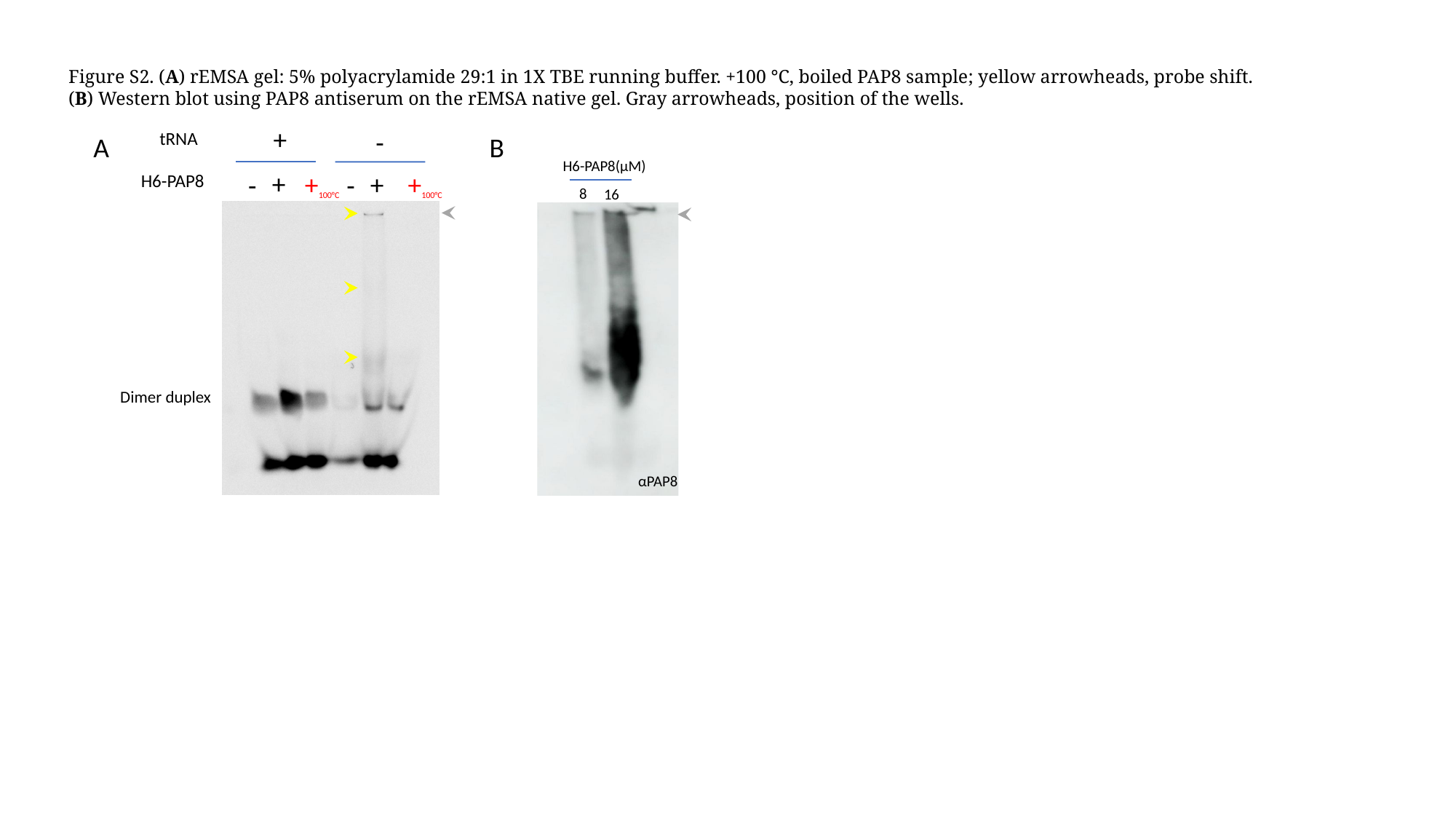

Figure S2. (A) rEMSA gel: 5% polyacrylamide 29:1 in 1X TBE running buffer. +100 °C, boiled PAP8 sample; yellow arrowheads, probe shift.
(B) Western blot using PAP8 antiserum on the rEMSA native gel. Gray arrowheads, position of the wells.
+
-
+
+100°C
+
+100°C
-
-
tRNA
H6-PAP8
A
B
H6-PAP8(µM)
8
16
Dimer duplex
αPAP8
